# Supplementary material for: Prokineticin-2 prevents neuronal cell deaths in a model of traumatic brain injury
Source: Nat Commun. 2021 Jul 9;12:4220. doi: 10.1038/s41467-021-24469-y (PMC8270965; doi:10.1038/s41467-021-24469-y)
Supplement: Supplementary file 1 — Supplementary information [file 41467_2021_24469_MOESM1_ESM.pdf]

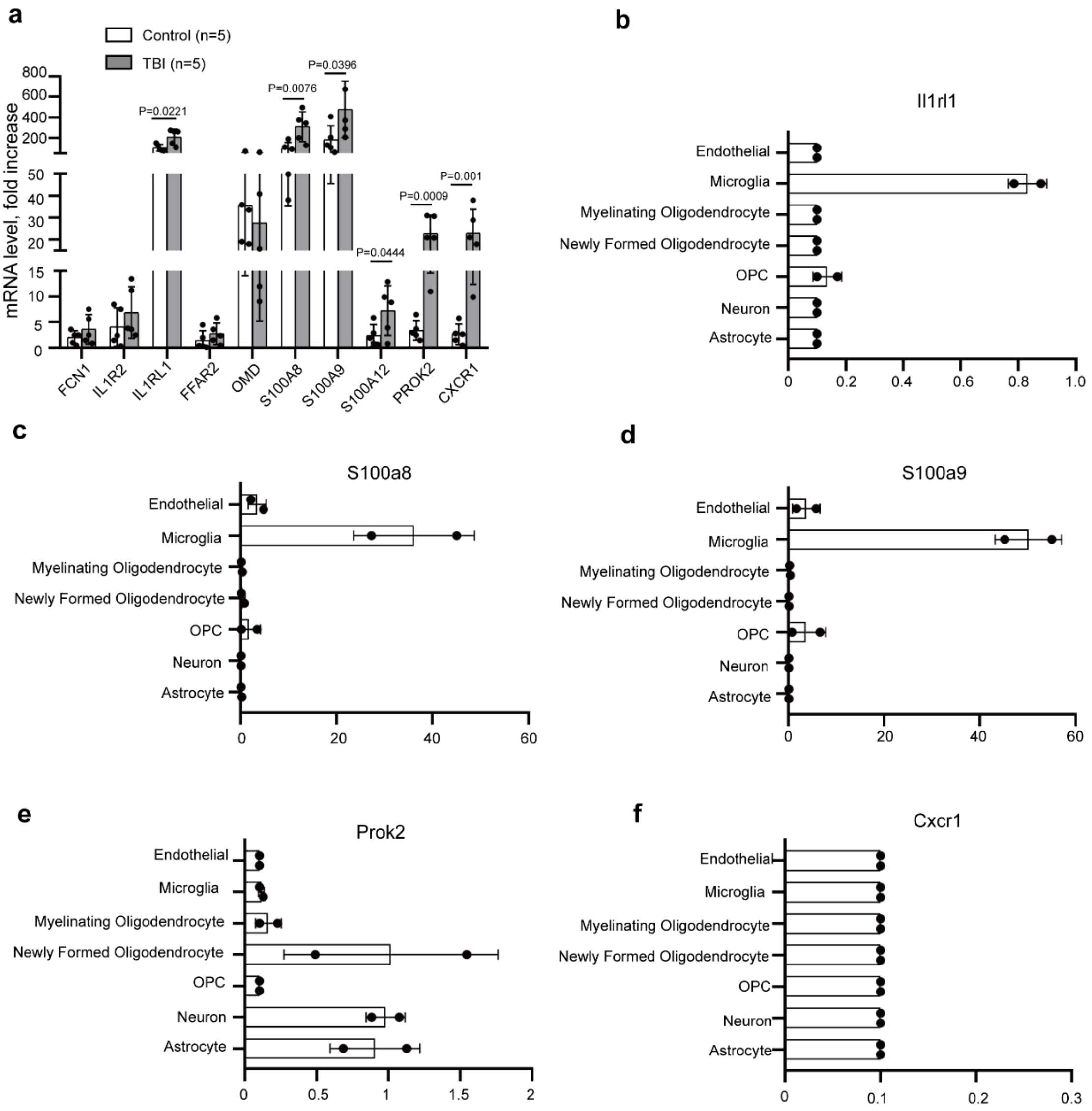

Supplementary Fig. 1 Analysis of selected genes. (**a**) qRT-PCR is used to confirm the mRNA difference between human control and TBI group. *IL1RL1*, *S100A8*, *S100A9*, *S100A12*, *PROK2* and *CXCR1* are obtained. * $P < 0.05$, ** $P < 0.01$, *** $P < 0.001$ versus respective control group. Error bars represent S.D. (n = 5 experiments). (**b**-**f**) Brain RNA Data base (GSE 52564) or [https://web.stanford.edu/group/barres_lab/brain_rnaseq.html] is used to test the expressions of five genes in brain. Data are presented as mean values ± S.D. (n = 2 samples). *Il1rl1* (**b**), *S100a8* (**c**) and *S100a9* (**d**) expressions are mainly in astrocyte but little in neuron; *S100a12* is not assayed in this data bank. (**f**) *Cxcr1* has a less expression and no difference in no matter cell styles. (**e**) *Prok2* has a high relative expression in neuron than the previous four, which means it is more highly selective

to neurons. For all panels, *n* indicates biologically independent repeats. *P* value was determined by two-tailed unpaired Student's *t* test for comparations between 2 groups. Source data are provided as a Source Data file.

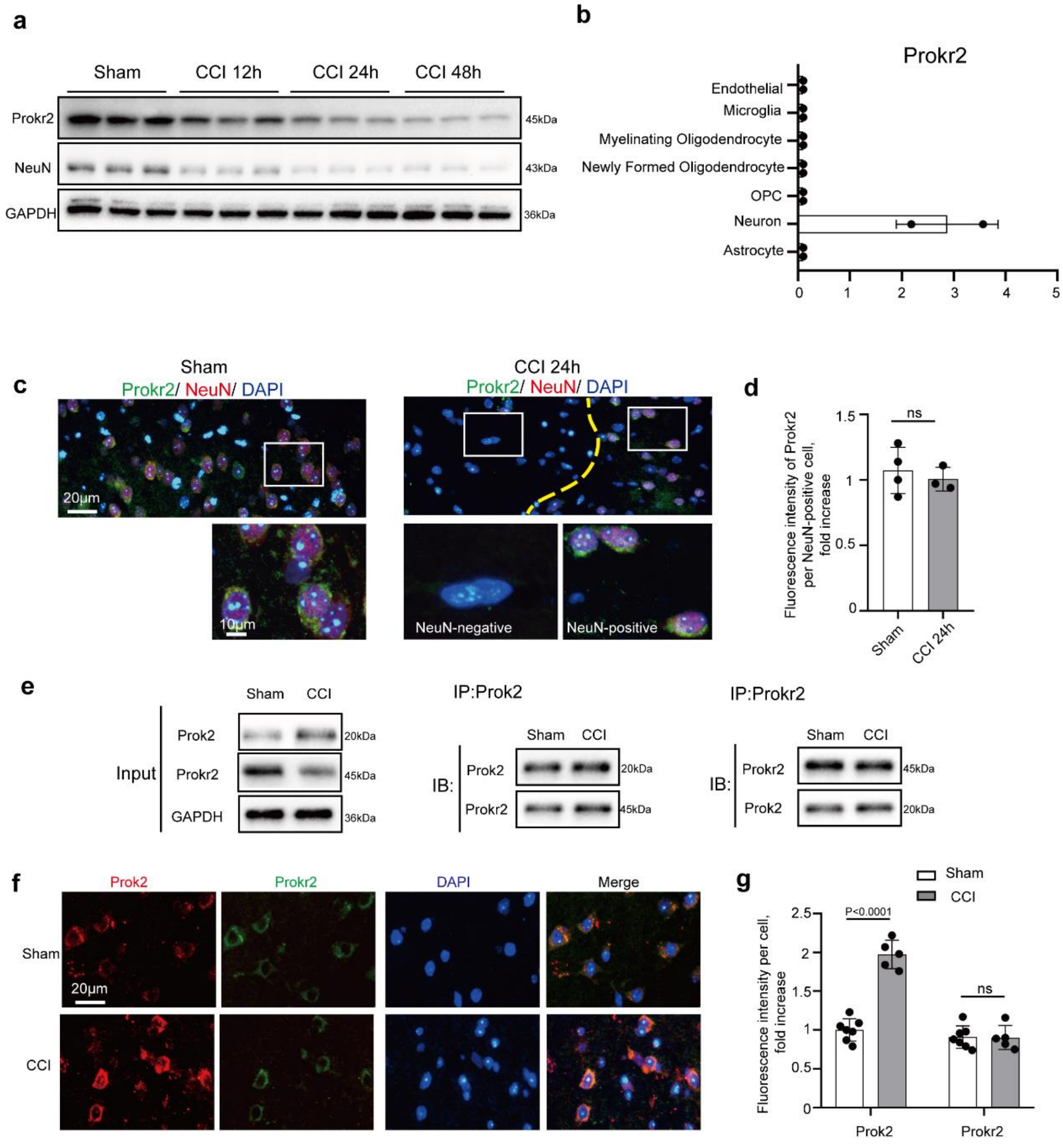

Supplementary Fig. 2 The level of Prokr2 in CCI. (**a**) Expression of Prokr2 and NeuN tested after CCI. GAPDH is used as a control (*n* = 3 mice per group). (**b**) Brain RNA Data base indicates that the Prokr2 expression is specifically located in neurons. Data are presented as mean values ± S.D. (*n* = 2 samples). (**c** and **d**)

Immunofluorescent staining assays show that Prokr2 is expressed predominantly in NeuN-positive cells. Scale bar is 20 μm and 10 μm. DAPI is used to stain cell nucleus. Prokr2 fluorescence intensity per NeuN-positive cell is measured by ImageJ. Error bars represent S.D. (*n* = 4 in sham group and *n* = 3 in CCI group). (**e**) Co-IP assays document that interactions between Prok2 and Prokr2 are not changed after CCI. Note the changes in the average expression of the two proteins in the brain tissue after CCI. (**f** and **g**) Dual immunofluorescence staining with Prok2 (red) and Prokr2 (green). Scale bar is 20 μm. Prok2 and Prokr2 fluorescence intensities per cell are measured by ImageJ. Data are presented as mean values ± S.D. (*n* = 7 in sham group and *n* = 5 in CCI group). For all panels, *n* indicates biologically independent repeats. *P* value was determined by two-tailed unpaired Student's *t* test for comparations between 2 groups. Source data are provided as a Source Data file.

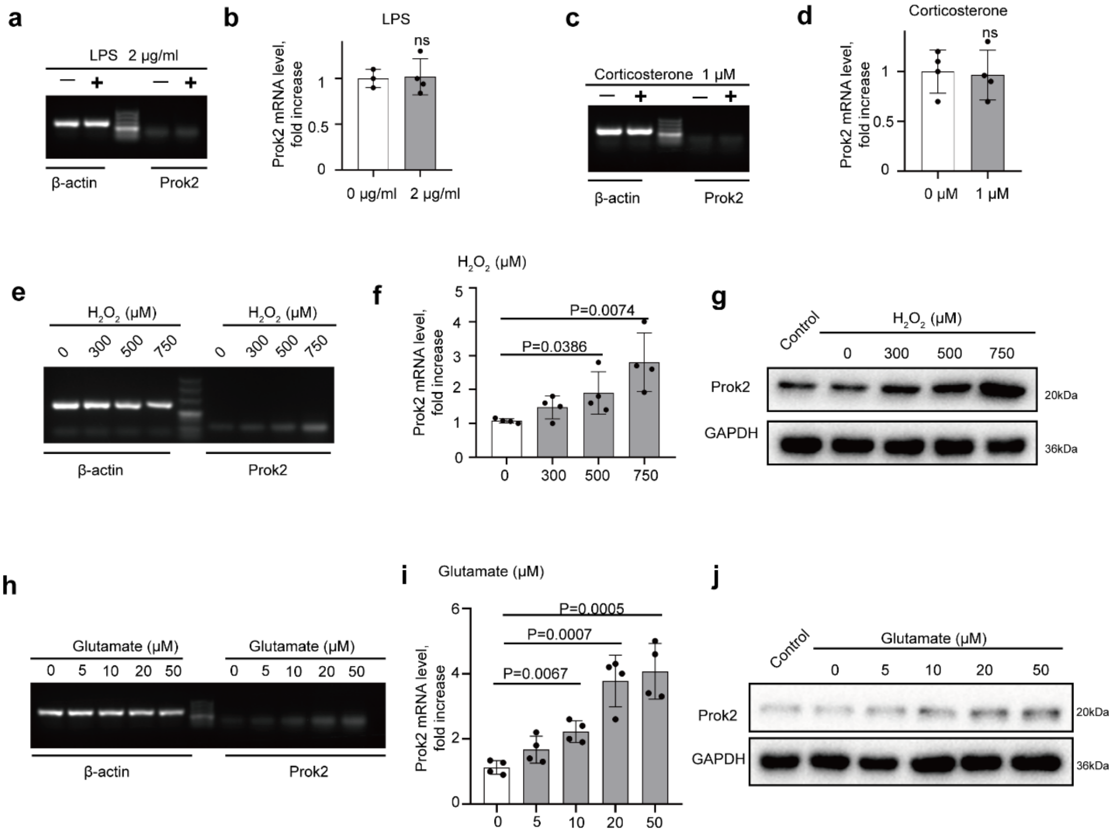

Supplementary Fig. 3 Prok2 is up-regulated by pathological insults including H_2O_2 and Glutamate *in vitro*. Neurons are treated with (**a**, **b**) LPS (2 ug/ml) for 24h, (**c**, **d**) corticosterone (1 µM) for 24h, (**e-g**) H_2O_2 for 12h, (**h-j**) excitotoxic levels of glutamate for 12h. *Prok2* mRNA is unchanged by LPS (P = 0.4913) and corticosterone (P = 0.7732). Glutamate and H_2O_2 increase *Prok2* mRNA and protein expression. *β-actin* is used as a control in semi-qRT-PCR assays. GAPDH is used as a control in western blot assays. Data are presented as mean values ± S.D. (n = 3 experiments). For all panels, n indicates biologically independent repeats. P value was determined by two-tailed unpaired Student's t test for comparations between 2 groups. Source data are provided as a Source Data file.

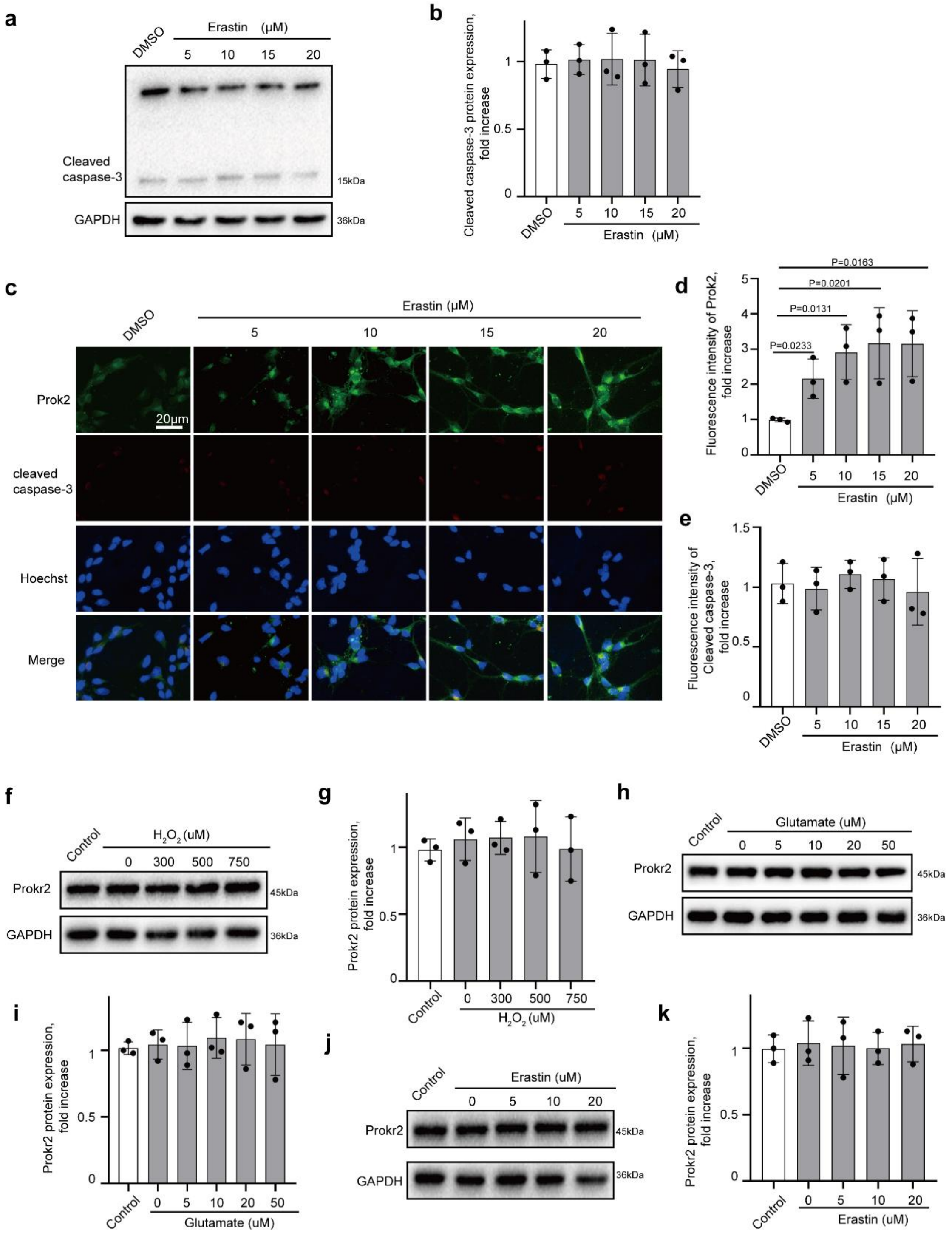

Supplementary Fig. 4 Erastin makes little impact on cleaved caspase-3 and Prokr2 is not sensitive to various pathological insults. (**a** and **b**) Erastin (within the concentration range from 5 to 20 μM), does not significantly affect levels of cleaved caspase-3 (GAPDH is used as a control). Data are presented as mean values ± S.D. (*n* = 3 experiments). (**c-e**) Immunostaining assays using dual-labeling of Prok2 (green) and cleaved caspase-3 (red). Scale bar is 20 μm. Hoechst is used to stain cell nuclei.

The fluorescence intensities of Prok2 and cleaved caspase-3 are measured by ImageJ. Data are presented as mean values ± S.D. (n = 3 experiments). (**f-k**) Western blot is used to test Prokr2 expression under H_2O_2 (**f-g**), Glutamate (**h-i**) and Erastin (**j-k**) stimulus. GAPDH is a control. Data are presented as mean values ± S.D. (n = 3 experiments). For all panels, n indicates biologically independent repeats. P value was determined by two-tailed unpaired Student's t test for comparations between 2 groups. Source data are provided as a Source Data file.

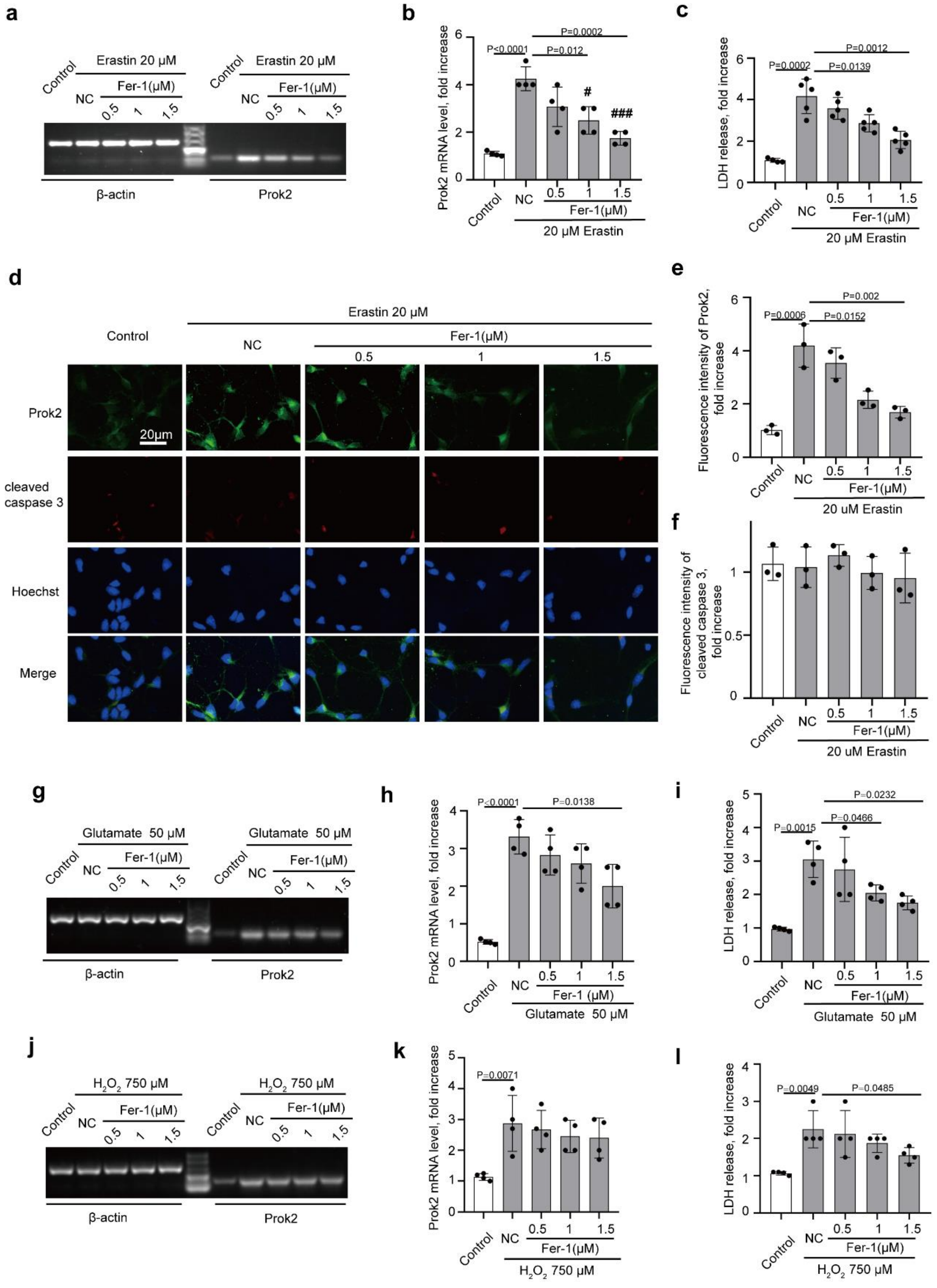

Supplementary Fig. 5 Fer-1 could inhibit the cytotoxicity of Erastin, Glutamate and H_2O_2. (**a-c**) Fer-1 decreases *Prok2* mRNA and LDH induced by Erastin. Data are presented as mean values ± S.D. (*n* = 3 experiments). (**d-f**) Dual-labeled immunofluorescence staining with Prok2 (green) and cleaved caspase-3 (red) is used

to detect their expressions after Fer-1 administration. Hoechst is used to stain cell nucleus. The fluorescence intensities are measured by ImageJ. Data are presented as mean values ± S.D. (*n* = 3 experiments). (**g**-**i**) Fer-1 decreases the up-regulated *Prok2* mRNA and LDH induced by excitotoxic glutamate. Data are presented as mean values ± S.D. (*n* = 3 experiments). (**j**-**l**) Fer-1 decreases the up-regulated LDH induced by H_2O_2, but does not change *Prok2* mRNA. Data are presented as mean values ± S.D. (*n* = 3 experiments). NC (negative control) refers to Erastin (20 μM) group. For all panels, *n* indicates biologically independent repeats. *P* value was determined by two-tailed unpaired Student's *t* test for comparations between 2 groups. Source data are provided as a Source Data file.

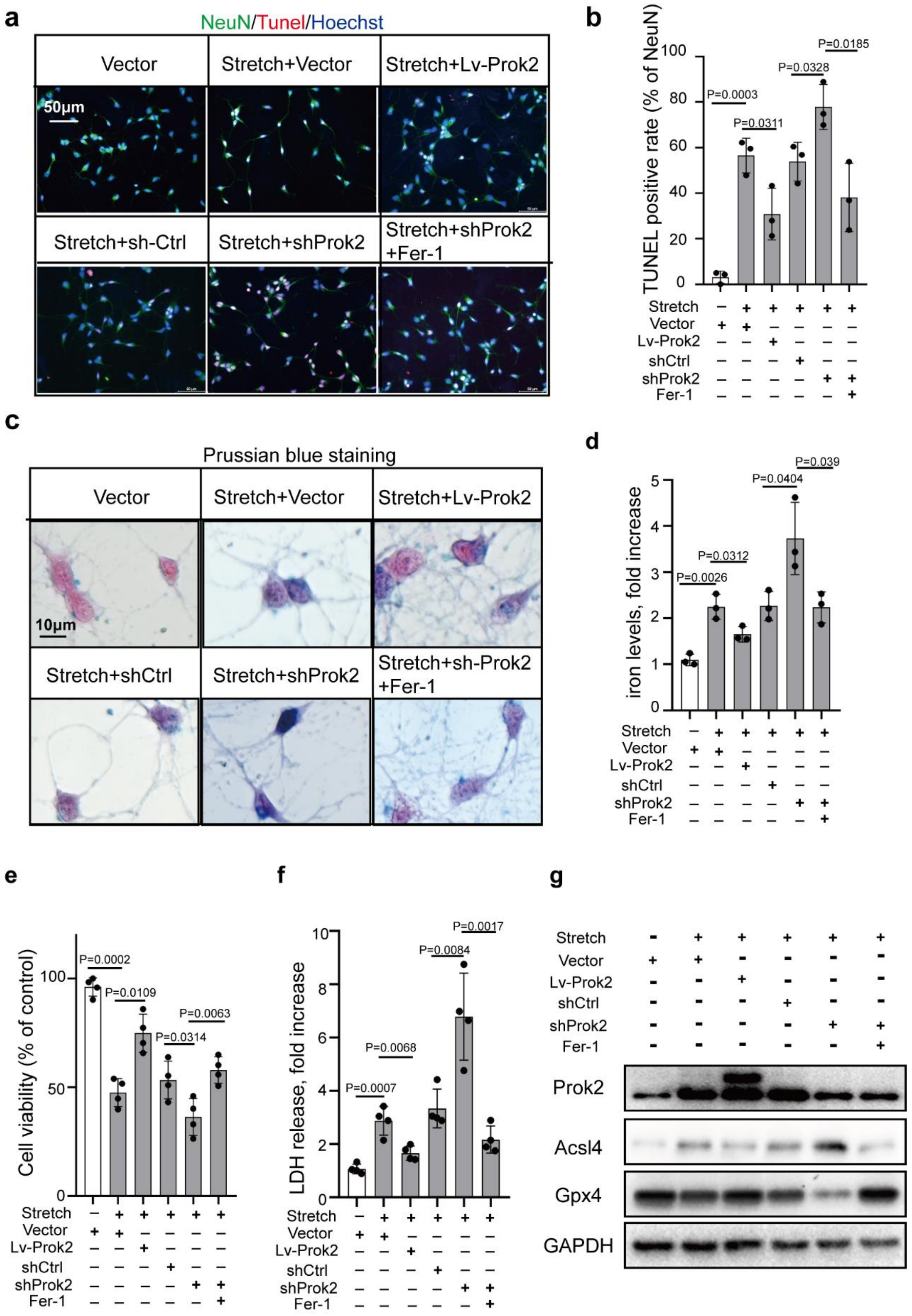

Supplementary Fig. 6 Overexpression of Prok2 attenuates stretch-induced cell death. Primary cortical neurons are exposed to stretch. The following characteristics are assessed at 6 h after stretch: (**a-b**) Cell death measured by TUNEL staining. Scale

bar is 50 μm. Data are presented as mean values ± S.D. (*n* = 3 experiments). (**c-d**) Mechanical stretch leads to increase in intracellular Fe3+ (blue) levels assessed by Prussian blue staining in primary cortical neurons. Lv-Prok2 attenuates this increase. Scale bar is 10 μm. Relative iron level is analyzed according to color intensity in ImageJ. Data are presented as mean values ± S.D. (*n* = 3 experiments). (**e-f**) Cell viability is analyzed by CCK-8. Cytotoxicity induced by stretch is measured by LDH assay. Data are presented as mean values ± S.D. (*n* = 4 experiments). (**g**) Expression of Prok2, Gpx4 and Acsl4 is normalized to GAPDH levels used as controls in western blot assays. For all panels, *n* indicates biologically independent repeats. *P* value was determined by two-tailed unpaired Student's *t* test for comparations between 2 groups. Source data are provided as a Source Data file.

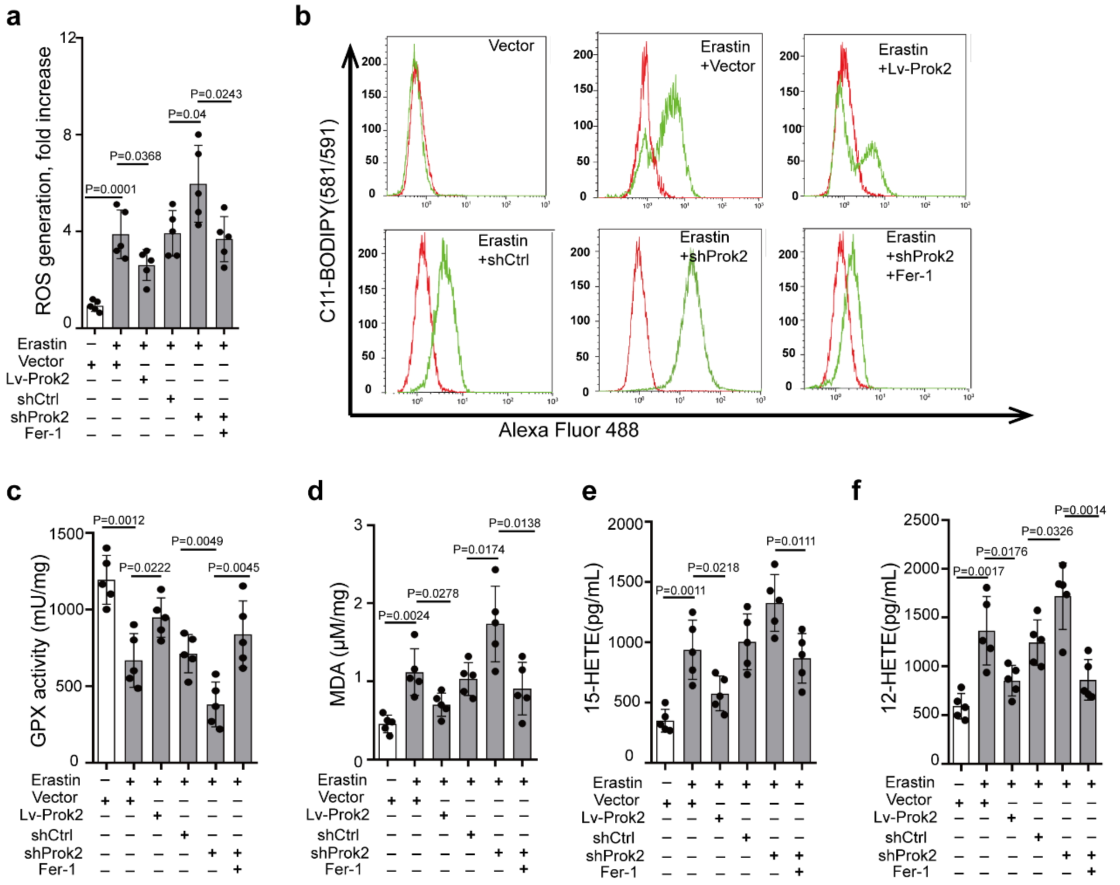

Supplementary Fig. 7 Prok2 upregulation decreases peroxidation process induced by Erastin. (**a**) ROS generation (**b**) Cell lipid peroxidation is detected by BODIPY 581/591 C11 staining using flow cytometer. (**c)** The level of GPX activity. (**d**) Lipid peroxidation tested by MDA assay. (**e**, **f**) 15-HETE and 12-HETE generation are also tested by corresponding kits. Data are presented as mean values ± S.D. (*n* = 5 experiments). For all panels, *n* indicates biologically independent repeats. *P* value was determined by two-tailed unpaired Student's *t* test for comparations between 2 groups. Source data are provided as a Source Data file.

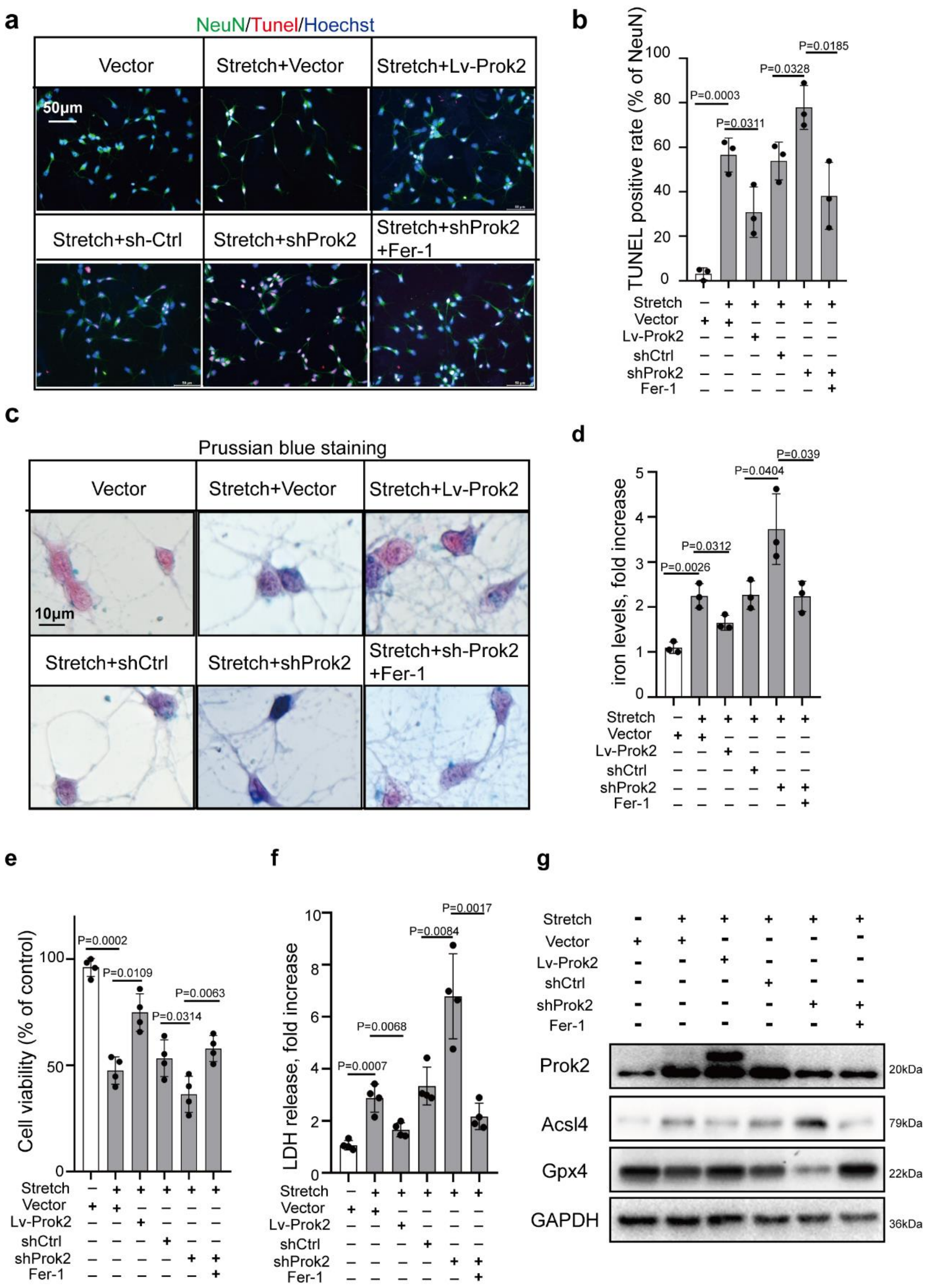

Supplementary Fig. 8 Prok2 overexpression alleviates stretch-induced lipid peroxidation. (**a**) ROS generation (**b-c**) Lipid peroxidation detected by BODIPY 581/591 C11 staining (green). Hoechst is used to stain cell nuclei. Scale bar is 50 μm.

The percentage of BODIPY 581/591 C11-positve cells is presented. (**d**) GPX activity. (**e**) Lipid peroxidation assessed by MDA assay. (**f, g**) 15-HETE and 12-HETE generation quantified by corresponding kits. Data are presented as mean values ± S.D. (*n* = 4 experiments); *n* = 3 experiments for measurements of BODIPY 581/591 C11-positive cells; *n* = 5 experiments for GPX activity assays. For all panels, *n* indicates biologically independent repeats. *P* value was determined by two-tailed unpaired Student's *t* test for comparations between 2 groups. Source data are provided as a Source Data file.

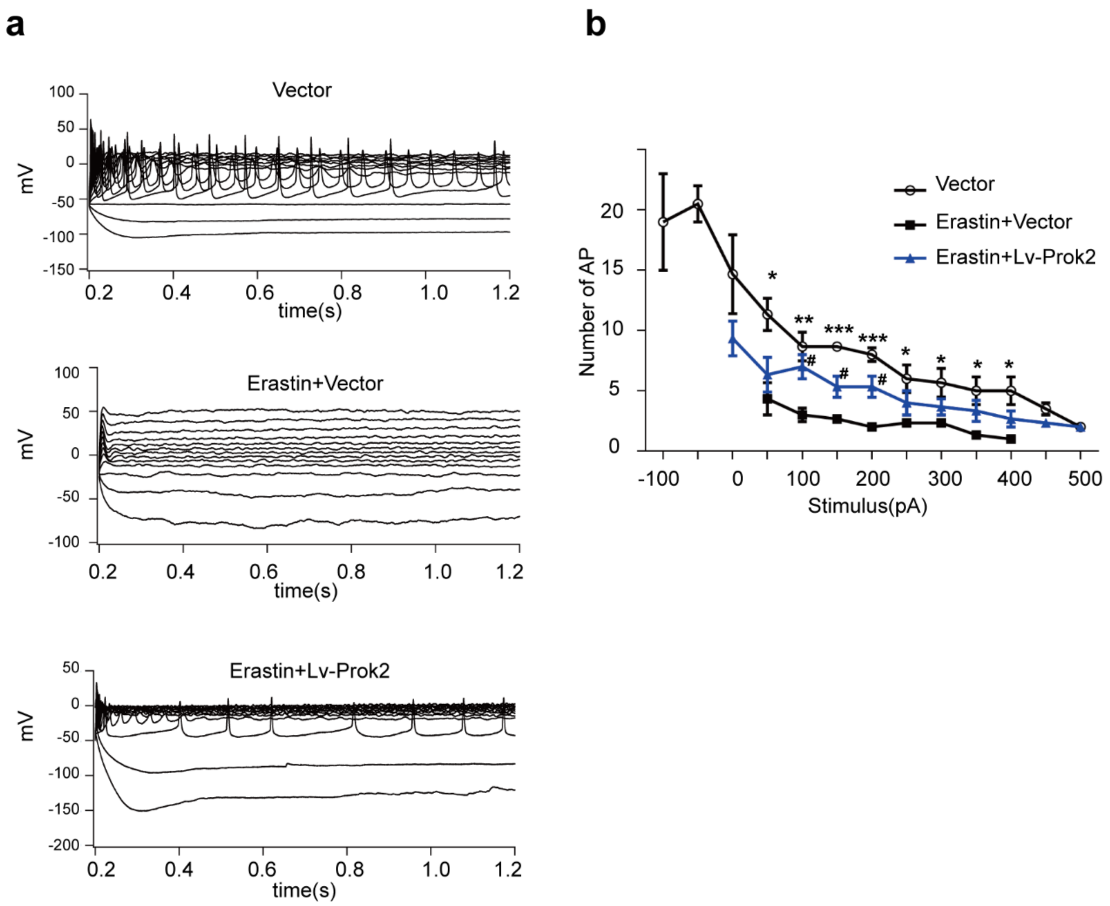

Supplementary Fig. 9 Neuronal electrophysiological activity test. (**a**) Neuronal action potentials (AP) are recorded to display physiological activity in various electricity stimulus. (**b**) The number of AP is measured. Data are presented as mean values ± S.E.M. (n = 3 experiments). * P <0.05, ** P <0.01 and *** P <0.01 versus vector group. $P_{(50pA)} = 0.0206$, $P_{(100pA)} = 0.0132$, $P_{(150pA)} = 0.0002$, $P_{(200pA)} = 0.0005$, $P_{(250pA)} = 0.038$, $P_{(300pA)} = 0.038$, $P_{(350pA)} = 0.038$ and $P_{(400pA)} = 0.0257$; $^{\#}$ P >0.05 versus vector + Erastin group. $P_{(100pA)} = 0.0257$, $P_{(150pA)} = 0.0474$ and $P_{(200pA)} = 0.0194$. An unpaired t-test and one-way analysis of variance (ANOVA) plus Tukey's test was used to analyze the data at each stimulus (pA). For all panels, n indicates biologically independent repeats. Source data are provided as a Source Data file.

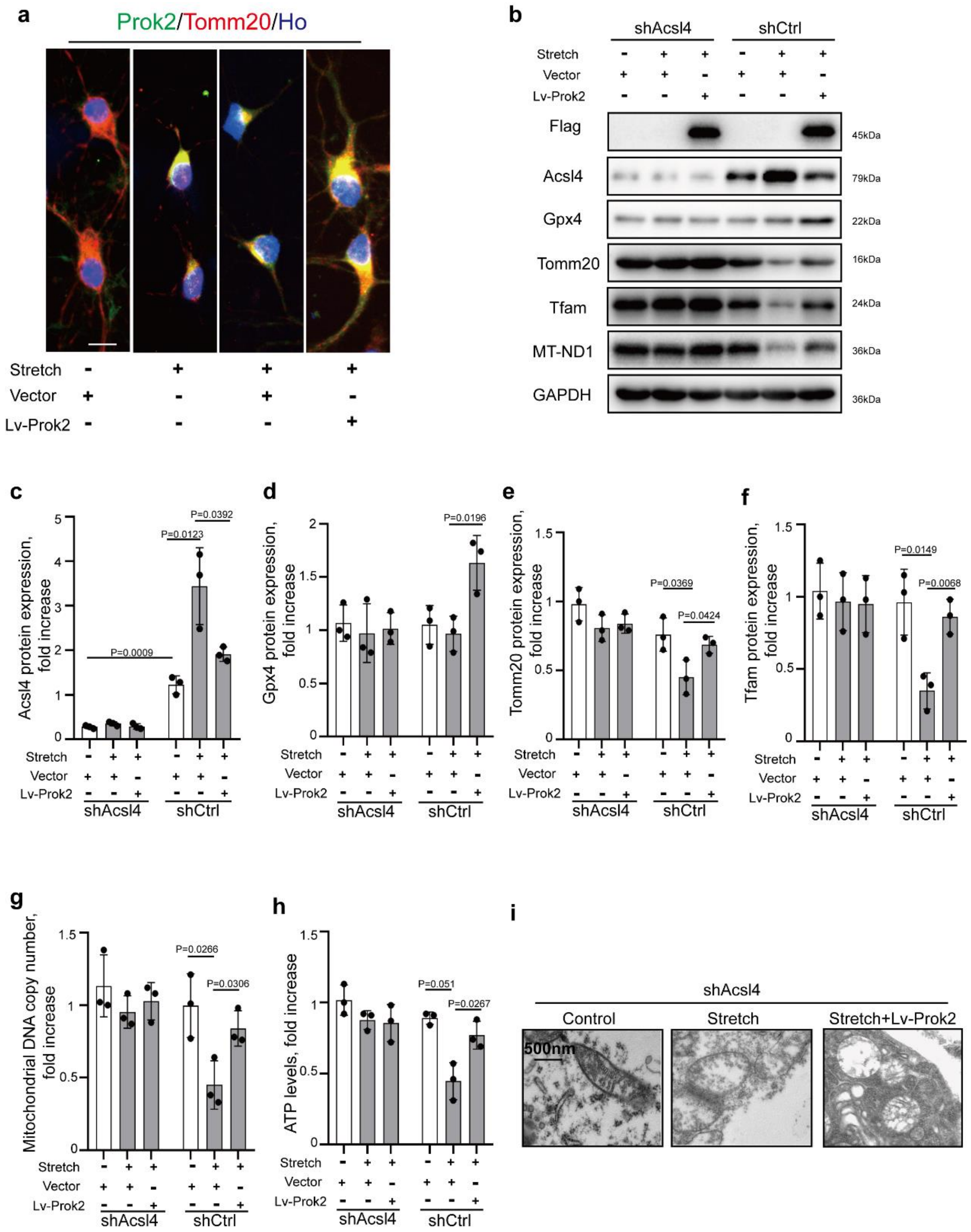

Supplementary Fig. 10 Prok2 protects mitochondrial function in stretch model in an Acsl4 dependent manner. (**a**) Expression and intracellular distribution of Tomm20 (red) and Prok2 (green) are shown by immunofluorescence. Hoechst stains cell nuclei. Scale bar is 15 μm. (**b-g**) Expressions of Acsl4, Gpx4, Tomm20, Tfam and MT-ND1 in Acsl4 deficient (shAcsl4) and control (shCtrl) primary neurons. GAPDH is used as a control. Data are presented as mean values ± S.D. (*n* = 3 experiments) in

c-g. (**h**) ATP levels are decreased upon stretch exposure. Overexpression of Prok2 prevents the decrease in ATP levels in stretched cells. Data are presented as mean values ± S.D. (*n* = 3 experiments). (**i**) Representative TEM images show that stretch does not cause mitochondrial shrinking in shAcsl4 primary neurons. Scale bar is 500 nm. For all panels, *n* indicates biologically independent repeats. *P* value was determined by two-tailed unpaired Student's *t* test for comparations between 2 groups. Source data are provided as a Source Data file.

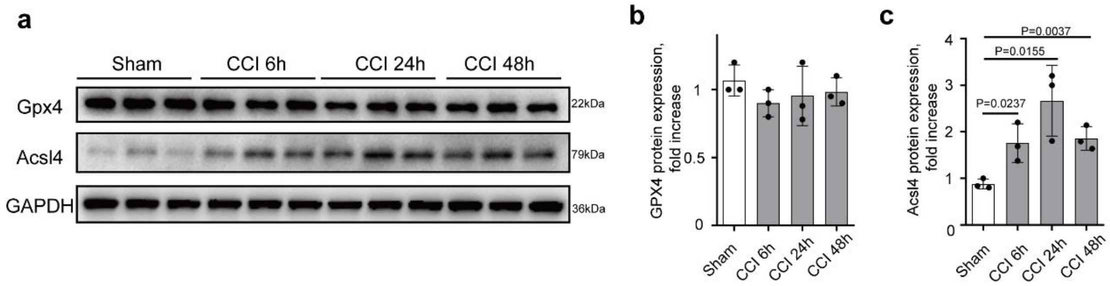

Supplementary Fig. 11 Expression of Acsl4 and Gpx4 in a CCI model. **a**, Western blot assays for Acsl4 and Gpx4. **b**, **c**, Acsl4 protein is increased after CCI. However, Gpx4 does not change significantly. Data are presented as mean values ± S.D. (n = 3 mice per group). For all panels, n indicates biologically independent repeats. P value was determined by two-tailed unpaired Student's t test for comparations between 2 groups. Source data are provided as a Source Data file.

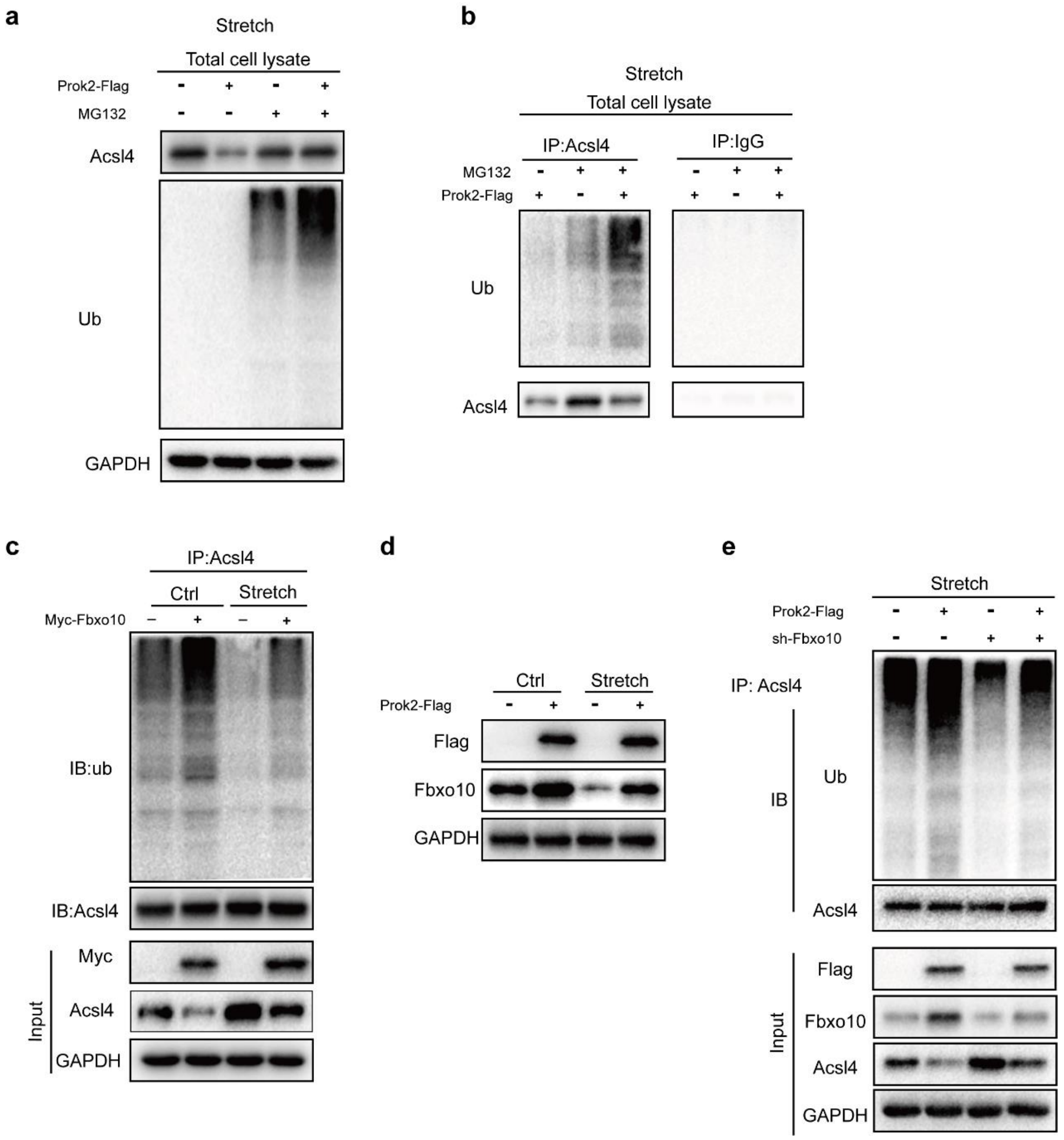

Supplementary Fig. 12 Prok2 promotes Acsl4 ubiquitination and degradation in stretch model. (**a**) Primary cortical neurons expressing control empty vector or Lv-Prok2 are treated with MG132 (20 μM) to block proteasomal degradation for 6 h and then exposed to mechanical stretch. Total lysates are analyzed for Acsl4 and ubiquitinated proteins by immunoblotting using anti-Acsl4 and anti-Ub. The decrease in Acsl4 protein observed upon Prok2 overexpression is abolished by MG132 and whereas ubiquitination of Acsl4 is increased. (**b**) IP with Acsl4 antibody and western blot with anti-Ub show that Acsl4 ubiquitination is higher in Prok2 overexpressing plus MG132 treated cells versus control untreated cells as well as empty vector

transfected plus MG132 treated cells. IgG is used as a negative control. (**c**) IP with Acsl4 followed by western blot with anti-Ub shows that overexpression of Fbxo10 increases Acsl4 ubiquitination in the presence or absence of stretch. (**d**) Overexpression of Prok2 in primary cortical neurons increases the level of Fbxo10 in the presence or absence of stretch. (**e**) Primary neurons are co-transfected with Lv-Prok2-Flag and Fbxo10 shRNA. Lysates of primary neurons exposed to stretch are immunoprecipitated with Acsl4 antibody followed by western blot with anti-Ub. Prok2-induced ubiquitination of Acsl4 is decreased in Fbxo10 deficient cells.

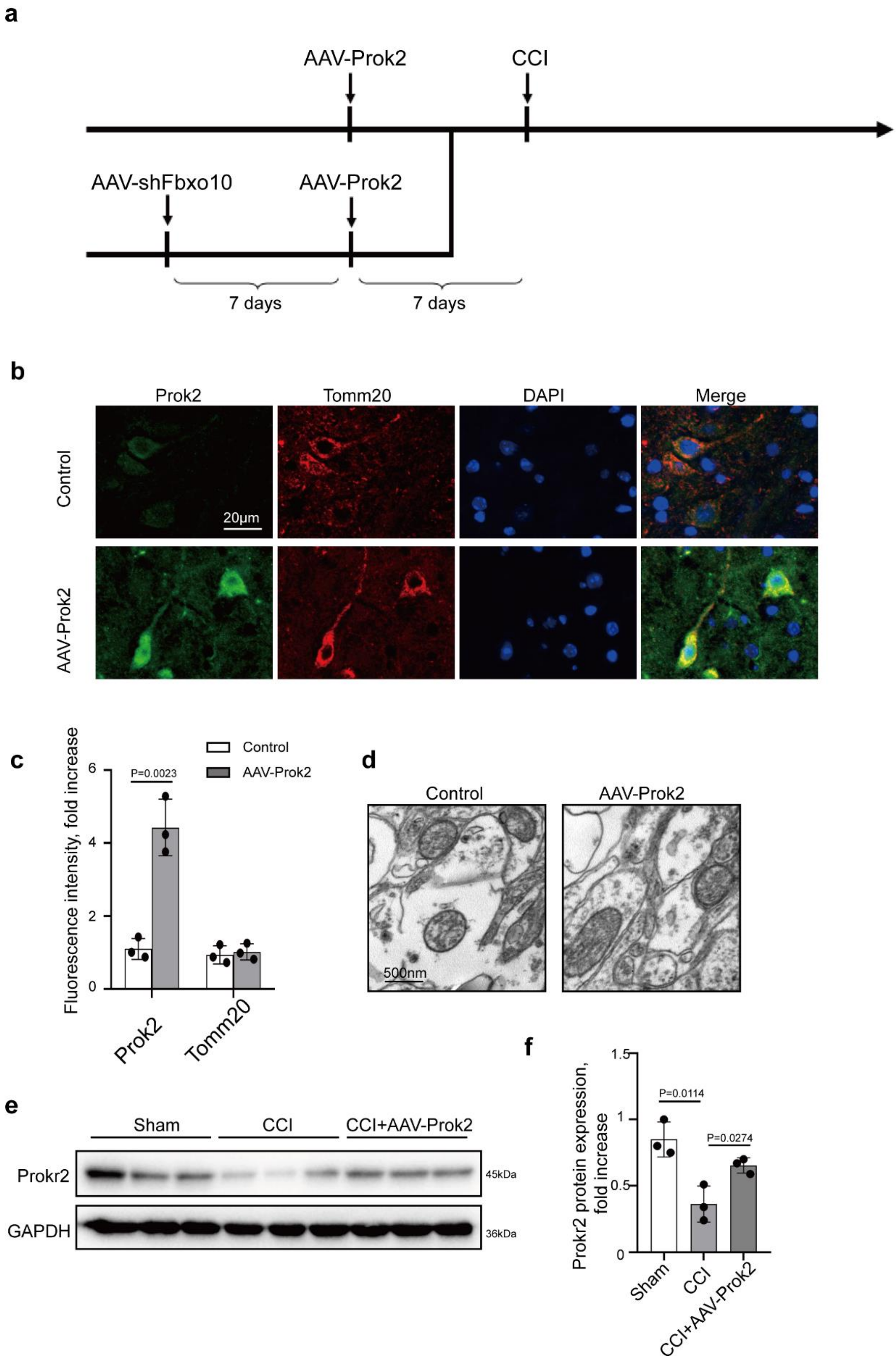

Supplementary Fig. 13 AAV-Prok2 and AAV-shFbxo10 are injected in mouse brain to perform transfection *in vivo*. (**a**) The schedule of intracranial gene and

shRNA delivery AAV-Prok2 or -shFbxo10 are designed for injection into mouse brain prior to CCI injury for effective transfection. At 7 days before CCI, AAV-Prok2 is injected. As for injection both AAV-Prok2 and -shFbxo10, the first injection, AAV-shFbxo10, is performed at 14 days before CCI. (**b-c**) Immunofluorescence staining of Tomm20 (red) and Prok2 (green) is used to test for possible effects of AAV injections on the mitochondria. No significant differences in Tomm20 staining between control and AAV-Prok2 are found. Scale bar is 20 μm. Data are presented as mean values ± S.D. (*n* = 3 mice per group) (*n* = 3 mice per group). (**d**) Representative TEM images of mitochondria in control and AAV-Prok2 groups. Scale bar is 500 nm. (**e-f**) Western blot assays documenting Prokr2 contents after Prok2 overexpression in CCI model. Data are presented as mean values ± S.D. (*n* = 3 mice per group). For all panels, *n* indicates biologically independent repeats. *P* value was determined by two-tailed unpaired Student's *t* test for comparations between 2 groups. Source data are provided as a Source Data file.

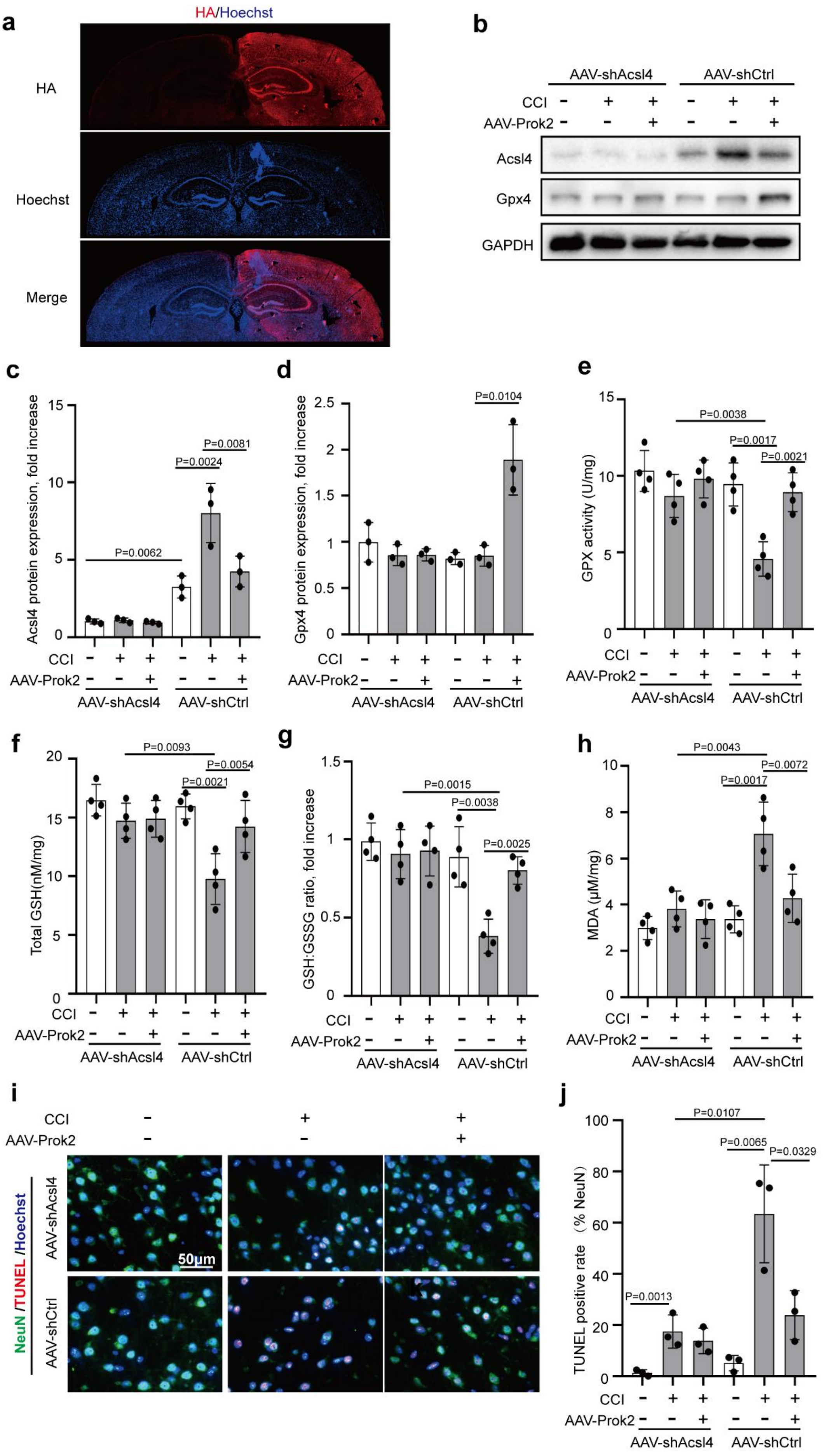
a
HA/Hoechst
HA
Hoechst
Merge
b
AAV-shAcsl4
AAV-shCtrl
CCI
AAV-Prok2
Acsl4
Gpx4
GAPDH
c
Acsl4 protein expression, fold increase
P=0.0062
P=0.0024
P=0.0081
d
Gpx4 protein expression, fold increase
P=0.0104
e
GPX activity (U/mg)
P=0.0038
P=0.0017
P=0.0021
f
Total GSH(nM/mg)
P=0.0093
P=0.0021
P=0.0054
g
GSH:GSSG ratio, fold increase
P=0.0015
P=0.0038
P=0.0025
h
MDA (μM/mg)
P=0.0043
P=0.0017
P=0.0072
i
NeuN /TUNEL /Hoechst
50μm
j
TUNEL positive rate （% NeuN）
P=0.0107
P=0.0065
P=0.0329
P=0.0013

Supplementary Fig. 14 Prok2 overexpression alleviates CCI-induced ferroptosis in an Acsl4 dependent manner. (**a**) AAV-shAcsl4 jointly with HA is injected into mouse brain prior to CCI. Immunostaining for HA is used to test the transfection efficiency. Hoechst staining of the nuclei is employed. (**b-d**) Western blot assays indicate that Acsl4 significantly declines after AAV-shAcsl4 treatment. GPX4 expression is not changed in Acsl4 knockdown group. Data are presented as mean values ± S.D. (n = 3 mice per group). (**e-j**) Overexpression of Prok2 prevents CCI-induced decrease in GPX activity, total GSH levels and the GSH/GSSG ratio and increase in MDA levels and the number of TUNEL and NeuN positive cells. These effects of Prok2 overexpression are lost in Acsl4 knock down group. Data are presented as mean values ± S.D. (n = 3 mice per group). For all panels, n indicates biologically independent repeats. P value was determined by two-tailed unpaired Student's t test for comparations between 2 groups. Source data are provided as a Source Data file.

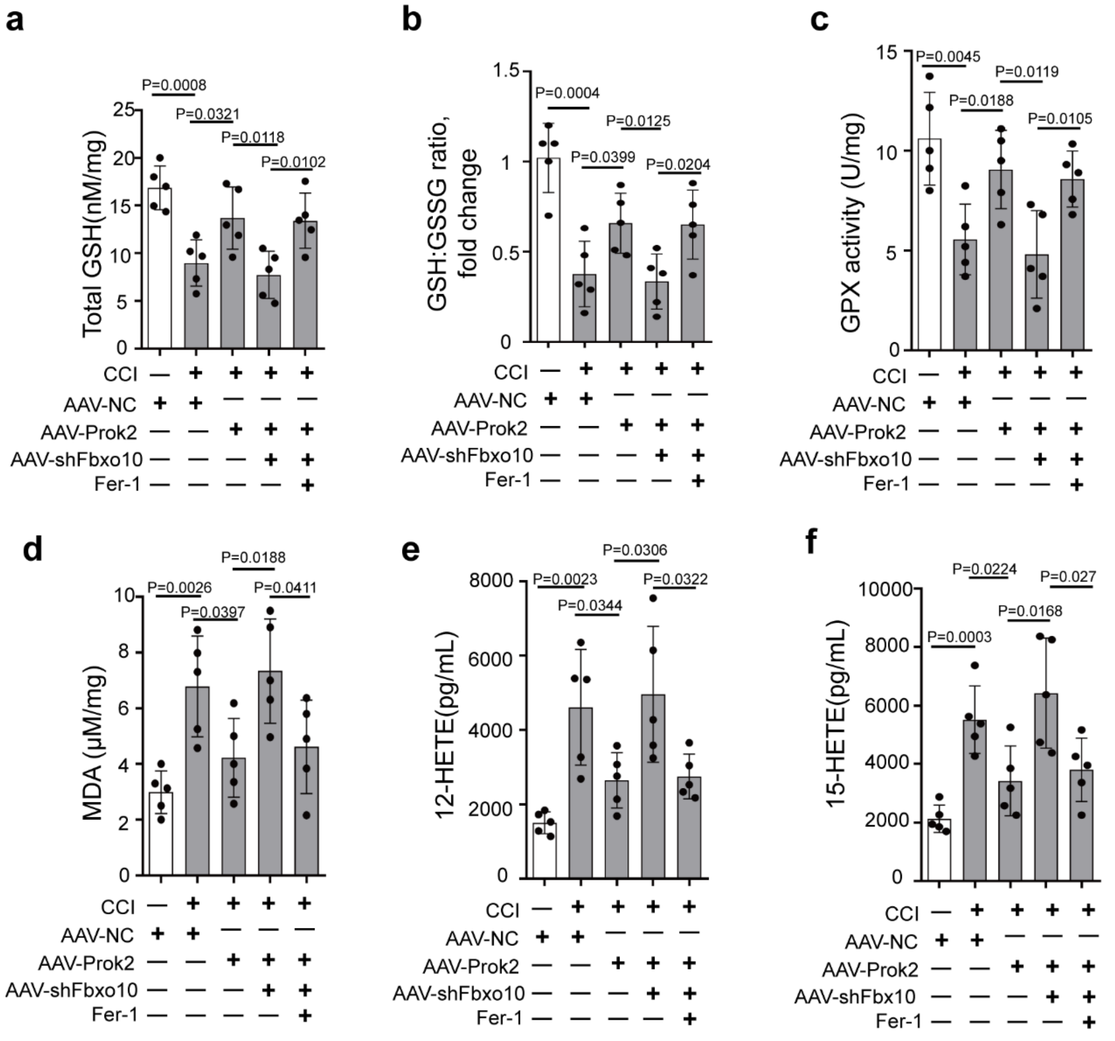

Supplementary Fig. 15 AAV-Prok2 intracerebroventricular injection could alleviate peroxidation induced by CCI. (**a** and **b**) Total GSH level and the GSH/GSSG ratio are detected. (**c**) GPX activity, (**d**) MDA and (**e**, **f**) the generation of 12-HETE and 15-HETE are detected by relevant kits. Data are presented as mean values ± S.D. (*n* = 5 mice per group). For all panels, *n* indicates biologically independent repeats. *P* value was determined by two-tailed unpaired Student's *t* test for comparations between 2 groups. Source data are provided as a Source Data file.

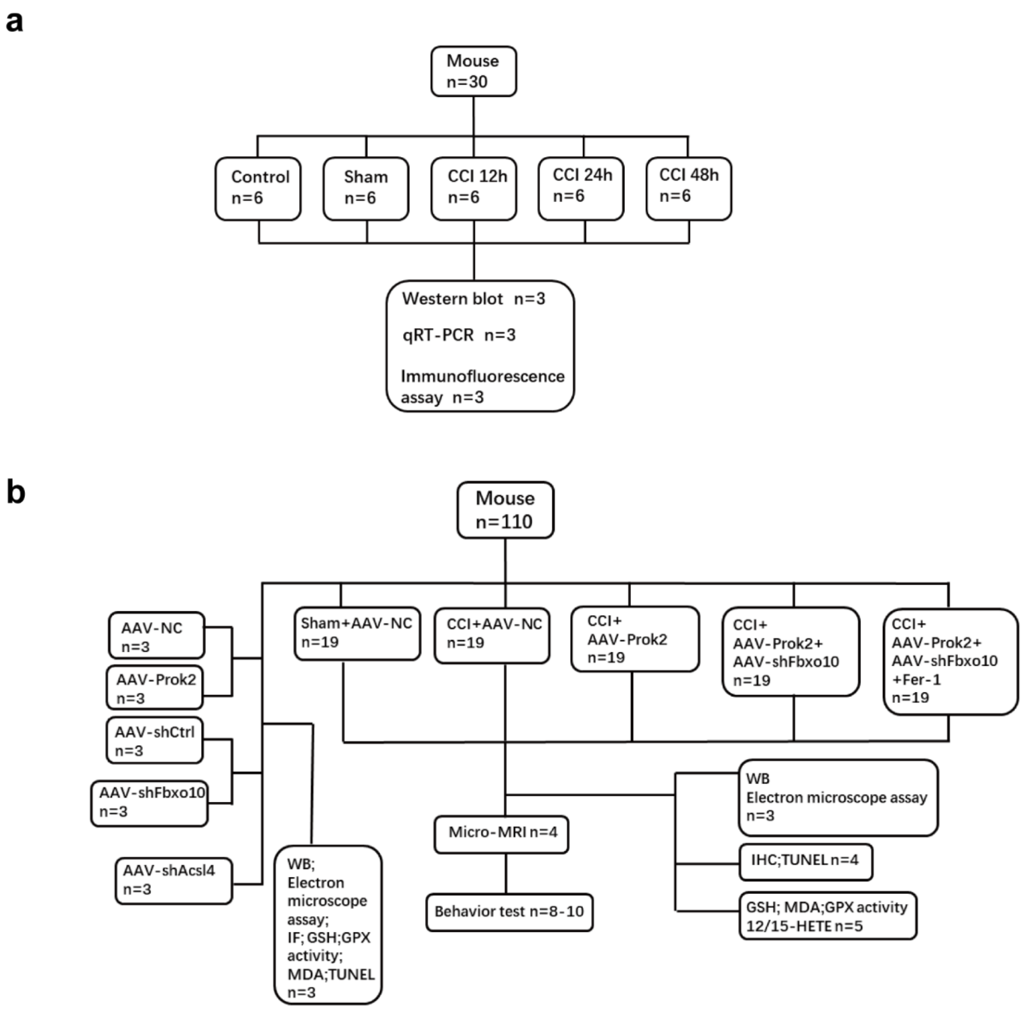

Supplementary Fig. 16 Schematic diagrams of animal experiment designs. (**a** and **b**) Schematic diagrams of animal experiment designs are shown, including each group with mice number and conducted experiments.

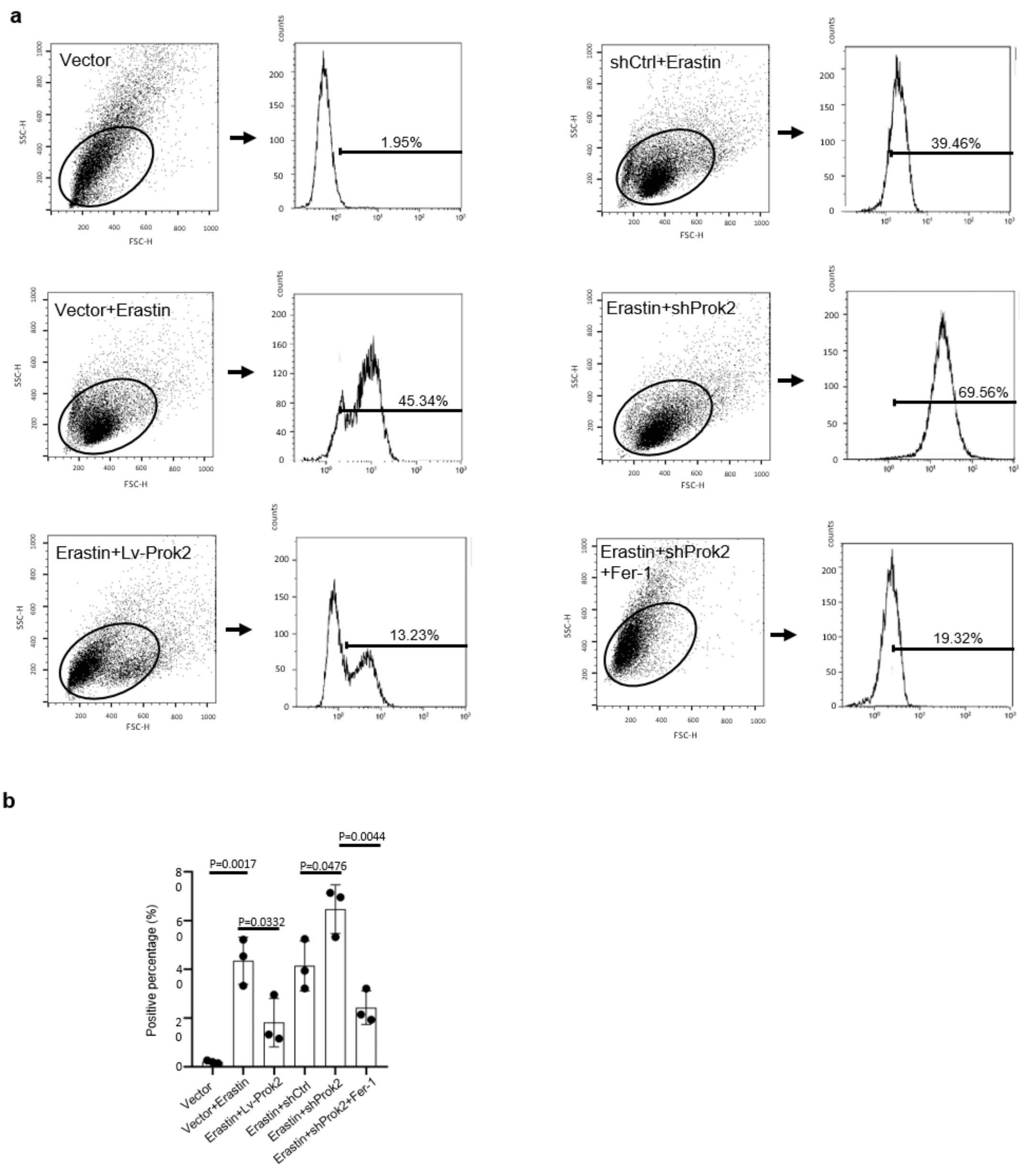

Supplementary Figure 17 FACS Sequential Gating Strategies. (**a**) Gating strategy to determine the percentage of cells with C11-BODIPY positive: 10,000 cells are analyzed per condition, the percentage in vector group is controlled within 5%, and then the percentage of the other groups are analyzed. (**b**) A bar graph shows the C11-BODIPY-positive rate in each group. The source data are provided as a Source Data file. Data are presented as mean values ± S.D. (n = 3). For all panels, n indicates biologically independent repeats. P value was determined by two-tailed unpaired Student's t test for comparations between 2 groups.

Supplementary Table 1 A complete list of all primers used.

Gene	Forward	Reverse
Prok2 (mouse)	CCTGAGGGGCTTGAAATTCAC	GTTGGTCTACTTGGAGGAACG
Acsl4 (mouse)	TCCTCCAAGTAGACCAACCCC	AGTCCAGGGATACGTTCACAC
Beta-actin (mouse)	GAGACCTTCAACACCCCAGC	ATGTCACGCACGATTTCCC
PROK2 (human)	TTCACACCCAACTTTAATCCACC	TCCATAGGGAGGTCATAATCACC
IL1R2 (human)	ACAGATGCTTTCCTGCCGTTC	ATCTTCCAGGGCCACATCGT
IL1RL1 (human)	GAGCACTTGCCAGTTTCCTC	CCTCCTGTTGTGCCTCTCTC
FFAR2 (human)	TTCCCCGTGCAGTACAAGCTC	CCACACAAAACGCCAGTAGCAG
OMD (human)	TAACCTCAGCCACAACAA	AGCCCATCCATAGCATTT
S100A8 (human)	CGACGTCTACCACAAGTACTCCC	GCCACGCCCATCTTTATCACC
S100A9 (human)	TCATCAACACCTTCCACCAA	TTAGCCTCGCCATCAGCA
S100A12 (human)	CTTCCACCAATACTCAGTTCGGA	CCTTCAGCGCAATGGCTACCAG
CXCR1 (human)	TTTGTTTGTCTTGGCTGCTG	AGTGTACGCAGGGTGAATCC
BETA-ACTIN (human)	CATCATCCCTGCCTCTACTG	GCCTGCTTCACCACCTTC
