# Supplementary material for: Prokineticin-2 prevents neuronal cell deaths in a model of traumatic brain injury
Source: Nat Commun. 2021 Jul 9;12:4220. doi: 10.1038/s41467-021-24469-y (PMC8270965; doi:10.1038/s41467-021-24469-y)
Supplement: Supplementary file 3 — Reporting Summary [file 41467_2021_24469_MOESM3_ESM.pdf]

nature research

Corresponding author(s): Jing Ji

Last updated by author(s): Jun 12, 2021

Reporting Summary

Nature Research wishes to improve the reproducibility of the work that we publish. This form provides structure for consistency and transparency in reporting. For further information on Nature Research policies, see our Editorial Policies and the Editorial Policy Checklist.

Statistics

For all statistical analyses, confirm that the following items are present in the figure legend, table legend, main text, or Methods section.

n/a	Confirmed	
☐	☒	The exact sample size (*n*) for each experimental group/condition, given as a discrete number and unit of measurement
☐	☒	A statement on whether measurements were taken from distinct samples or whether the same sample was measured repeatedly
☐	☒	The statistical test(s) used AND whether they are one- or two-sided<br>*Only common tests should be described solely by name; describe more complex techniques in the Methods section.*
☒	☐	A description of all covariates tested
☐	☒	A description of any assumptions or corrections, such as tests of normality and adjustment for multiple comparisons
☐	☒	A full description of the statistical parameters including central tendency (e.g. means) or other basic estimates (e.g. regression coefficient) AND variation (e.g. standard deviation) or associated estimates of uncertainty (e.g. confidence intervals)
☐	☒	For null hypothesis testing, the test statistic (e.g. *F*, *t*, *r*) with confidence intervals, effect sizes, degrees of freedom and *P* value noted<br>*Give P values as exact values whenever suitable.*
☒	☐	For Bayesian analysis, information on the choice of priors and Markov chain Monte Carlo settings
☒	☐	For hierarchical and complex designs, identification of the appropriate level for tests and full reporting of outcomes
☒	☐	Estimates of effect sizes (e.g. Cohen's *d*, Pearson's *r*), indicating how they were calculated

Our web collection on statistics for biologists contains articles on many of the points above.

Software and code

Policy information about availability of computer code

Data collection	Image Lab 5.2.1; SkanIt 6.0; Leica LAS AF Lite 2.6.0; Nanodrop 2000; TOPSCAN G3; ANY-MAZE 6.0.; Bruker Biospin MRI 1.34.2.1.; iTEM software 2.1.0(Olympus Soft Imaging Solutions, Münster, Germany).
Data analysis	ImageJ 1.8.0; Graphpad Prism 8.0, FlowJo V10.4, 3D slicer 4.11.20210226.

For manuscripts utilizing custom algorithms or software that are central to the research but not yet described in published literature, software must be made available to editors and reviewers. We strongly encourage code deposition in a community repository (e.g. GitHub). See the Nature Research guidelines for submitting code & software for further information.

Data

Policy information about availability of data

All manuscripts must include a data availability statement. This statement should provide the following information, where applicable:

- Accession codes, unique identifiers, or web links for publicly available datasets
- A list of figures that have associated raw data
- A description of any restrictions on data availability

The authors declare that all data supporting the findings of this study are available within this article, its supplementary information files, and source data. The source data underlying Figures 1-7 and Supplementary Figures 1-15 are provided as a Source Data file. The data of RNA-sequencing generated in this study have been deposited in the SRA database under accession code PRJNA730111 [https://www.ncbi.nlm.nih.gov/sra/PRJNA730111]. The brain data base [https://web.stanford.edu/group/barres_lab/brain_rnaseq.html] or GSE 52564 was used to identify the cell types where genes were mainly located. The datasets generated during and/or analyzed during the current study are available from the corresponding author on reasonable request.

Field-specific reporting

Please select the one below that is the best fit for your research. If you are not sure, read the appropriate sections before making your selection.

☒ Life sciences ☐ Behavioural & social sciences ☐ Ecological, evolutionary & environmental sciences

For a reference copy of the document with all sections, see nature.com/documents/nr-reporting-summary-flat.pdf

Life sciences study design

All studies must disclose on these points even when the disclosure is negative.

Sample size	Sample sizes were chosen based on preliminary data demonstrating statistically significant differences or previously reported data for each specific assay. For the cell viability test, MDA assay and GSH assay, we chose the sample sizes according to Yongfei Yang's study (PMID:31974380).
Data exclusions	No data were excluded from the analysis.
Replication	All experiments were performed with at least three technical replicates on more than one occasion to ensure reproducibility across experiments and all attempts at replication were successful.
Randomization	Samples and organisms were allocated into experimental groups randomly. All primary neuronal cells were obtained from new born mice and cultured at the same condition according to the protocol described in manuscript. All experimental treatments were controlled by the same dose, same reagent and same operation. The same method to collect samples in each experiment was used.
Blinding	The investigators were blinded to group allocation during data collection and analysis.

Reporting for specific materials, systems and methods

We require information from authors about some types of materials, experimental systems and methods used in many studies. Here, indicate whether each material, system or method listed is relevant to your study. If you are not sure if a list item applies to your research, read the appropriate section before selecting a response.

Materials & experimental systems

n/a	Involved in the study	
☐	☒	Antibodies
☐	☒	Eukaryotic cell lines
☒	☐	Palaeontology and archaeology
☐	☒	Animals and other organisms
☐	☒	Human research participants
☒	☐	Clinical data
☒	☐	Dual use research of concern

Methods

n/a	Involved in the study	
☒	☐	ChIP-seq
☐	☒	Flow cytometry
☒	☐	MRI-based neuroimaging

Antibodies

Antibodies used	Anti-Prok2 antibody, Abcam, Cat# ab76747, Rabbit polyclonal, Lot# GR284866-6; Anti-MT-ND1 antibody, Abcam, Cat# ab181848, Rabbit monoclonal [EPR13466(2)]. Anti-Fbx10, Novus, Cat# NBP1-91889, Rabbit polyclonal, Lot # R57903; Anti-Ubiquitin antibody, Abcam, Cat# ab19247, Rabbit polyclonal, Lot# GR185721-8; Anti-NeuN antibody, Abcam, Cat# ab279296,Mouse monoclonal [EPR12763], Lot# GR234241-2; Anti-Acsl4 antibody, Santa Cruz, Cat# sc-365230, Mouse monoclonal, Lot# 234243; Anti-Gpx4 antibody, Santa Cruz, Cat# sc-166570, Mouse monoclonal, Lot# 456353; Anti-Tomm20 antibody, Abcam, Cat# ab56783, Mouse monoclonal [4F3], Lot# GR 345324-2; Anti-Tfam antibody, Abcam, Cat# ab131607, Rabbit polyclonal, Lot# GR366434-1; Anti-GAPDH, YI FEI XUE BIOTECH, Cat# YFMA0037, Mouse monoclonal, Lot# 342311; Anti-Flag antibody, Beyotime, Cat# AF519, Mouse monoclonal, Lot# SR5631; Anti-HA, Beyotime, Cat# AH158, Mouse monoclonal, Lot# SR4234. Horseradish peroxidase-conjugated goat anti-rabbit IgG, Beyotime, Cat# A0208, Lot# SR3253; Horseradish peroxidase-conjugated goat anti-mouse IgG, Beyotime, Cat# A0216, Lot# SR2263. Alexa Fluor 488-conjugated secondary antibody, Jackson, Goat anti-mouse, Lot# 115-545-003; CyTM3-conjugated secondary antibody, Jackson, Goat anti-rabbit, Lot# 111-165-003. HRP-conjugated secondary antibody, Goat anti-Rabbit, Abcam, Cat# ab6112, Lot# GR345253-2.
Validation	Anti-Prok2 antibody, citation: Cells 8:N/A (2019) (PMID: 31766244). Manufacture's website: https://www.abcam.cn/prokineticin-2pk2-antibody-ab76747.html; Anti-MT-ND1, citation: Mitochondrion 54:57-64 (2020) (PMID: 32659360). Manufacture's website: https://www.abcam.cn/mt-nd1-antibody-epr134662-ab181848.html;Anti-Fbxo10 antibody. Manufacture's website: https://www.novusbio.com/products/fbx10-antibody_nbp1-91889#datasheet; Anti-Ubiquitin antibody, citation: Nat Methods 16:862-865 (2019) (PMID: 31471614). Manufacture's website:https://www.abcam.cn/ubiquitin-antibody-ab19247.html; Anti-NeuN antibody, manufacture's website:https://www.abcam.cn/neun-antibody-epr12763-ab279296.html; Anti-Acsl4 antibody, citation: Cell Rep 35: 109076 (2021) (PMID: 33951438). Manufacture's website: https://www.scbt.com/p/acsl4-antibody-f-4?requestFrom=search; Anti-

Gpx4 antibody, citation: Cell Death Dis. 10: 682 (2019) (PMID: 31527591). Manufacture's website: https://www.scbt.com/zh/p/gpx-4-antibody-e-12?requestFrom=search; Anti-Tomm20 antibody, citation:Redox Biol 28:101365 (2020) (PMID: 31707354). Manufacture's website: https://www.abcam.cn/tomm20-antibody-4f3-bsa-and-azide-free-ab56783.html; Anti-Tfam antibody, citation: Oncogene 39:617-636 (2020) (PMID: 31527668). Manufacture website: https://www.abcam.cn/mttfa-antibody-mitochondrial-marker-ab131607.html; Anti-GAPDH antibody, manufacture's website: http://www.yfxbio.com/product_info.asp?id=24370; Anti-Flag antibody, citation: Cell Death Dis 13;5:e1055 (2014) (PMID: 24525731).Manufacture's website: https://www.beyotime.com/product/AF519.htm; Anti-HA antibody, citation: Cell Res 22(2):333-45 (2012) (PMID: 21844891). Manufacture's website: https://www.beyotime.com/product/AH158.htm; Horseradish peroxidase-conjugated goat anti-rabbit IgG, citation: Brain Res 4;1494:1-8 (2013)(PMID:23219579). Manufacture's website: https://www.beyotime.com/product/AH158.htm; Horseradish peroxidase-conjugated goat anti-mouse IgG, citation: Brain Res 1311:189-96 (2010) (PMID: 19879861). Manufacture's website: https://www.beyotime.com/product/A0216.htm. Alexa Fluor 488-conjugated secondary antibody, citation: EBioMedicine 64:103213 (2021) (PMID:33508745). Manufacture's website: https://www.jacksonimmuno.com/catalog/products/115-545-003; CyTM3-conjugated secondary antibody, citation: Redox Biol,38:101830 (2021) (PMID:33338921). Manufacture's website: https://www.jacksonimmuno.com/catalog/products/111-165-003. HRP-conjugated Goat anti-Rabbit secondary antibody, citation: Exp Ther Med 19:2949-2956 (2020) (PMID: 322256780). Manufacture's website: https://www.abcam.cn/goat-fab2-rabbit-igg-fab2-hrp-preadsorbed-ab6112.html.

Eukaryotic cell lines

Policy information about cell lines

Cell line source(s)	Cells are isolated from the brain tissues of newborn mice.
Authentication	The primary neurons are identified via immunofluorescent assay with anti-NeuN and anti-Map2.
Mycoplasma contamination	We confirm the primary neurons tested for negative mycoplasma contamination.
Commonly misidentified lines (See ICLAC register)	Primary neurons are used in our manuscript.

Animals and other organisms

Policy information about studies involving animals; ARRIVE guidelines recommended for reporting animal research

Laboratory animals	Eight-week-old male C57BL/6 mice (Animal Core Facility of Nanjing Medical University, Nanjing, China). They were kept in an SPF condition with a 12h light/ 12h dark circle, 20~25℃ ambient temperature and 40%~70% humidity.
Wild animals	The study did not involve wild animals.
Field-collected samples	The study did not involve samples collected from the field.
Ethics oversight	Animal experiments were approved by the committee on Animal Care of Nanjing Medical University and performed in accordance with institutional guidelines.

Note that full information on the approval of the study protocol must also be provided in the manuscript.

Human research participants

Policy information about studies involving human research participants

Population characteristics	Designation Control1. Gender and age: Male, 34; Diagnosis: Arterial aneurysm; Treatment: Surgery.<br>Designation Control2. Gender and age: Female, 58; Diagnosis: Multiple cerebrovascular diseases; Treatment: Surgery.<br>Designation Control3. Gender and age: Female, 42; Diagnosis: Multiple cerebrovascular diseases; Treatment: Surgery.<br>Designation TBI1. Gender and age: Male, 42; Diagnosis: TBI (Left temporal lobe); Treatment: Surgery.<br>Designation TBI2. Gender and age: Female, 70; Diagnosis: TBI (Occipital lobe); Treatment: Surgery.<br>Designation TBI3. Gender and age: Male, 52; Diagnosis: TBI (Right temporal lobe); Treatment: Surgery.<br>Designation TBI4. Gender and age: Female, 58; Diagnosis: TBI (Left temporal lobe and occipital lobe); Treatment: Surgery.<br>Designation TBI5. Gender and age: Female, 58; Diagnosis: TBI (Left parietal lobe); Treatment: Surgery.
Recruitment	The tissue bank of the First Affiliated Hospital of Nanjing Medical University is established under a broad consent allowing for use of tissues and accompanying clinical information for the purposes of future research. Tissue in excess of that needed for diagnosis was allocated to the Bank. Bank recruitment covers all ages and a variety of diagnoses. Brain tissues in the 3 control groups were obtained during the surgical approach of arterial aneurysm and multiple cerebrovascular diseases. Traumatic brain tissues in the 5 TBI groups were obtain from patients diagnosed as TBI during surgery for removing intracranial hematoma. There is no obvious self-selection bias for the recruitment of these patients.
Ethics oversight	The use of human brain tissues was approved by the Research Ethics Committee of the First Affiliated Hospital of Nanjing Medical University (No. 2014-SRFA-106). Informed consents were obtained from the next of kin or patients. We declare that all methods used in this article were carried out in accordance with relevant guidelines and regulations of the First Affiliated Hospital of Nanjing Medical University.

Note that full information on the approval of the study protocol must also be provided in the manuscript.

Flow Cytometry

Plots

Confirm that:

- [x] The axis labels state the marker and fluorochrome used (e.g. CD4-FITC).
- [x] The axis scales are clearly visible. Include numbers along axes only for bottom left plot of group (a 'group' is an analysis of identical markers).
- [x] All plots are contour plots with outliers or pseudocolor plots.
- [x] A numerical value for number of cells or percentage (with statistics) is provided.

Methodology

Sample preparation	Depending on the experiment, primary neuronal cells were treated as defined in the relevant materials and methods. And cells were incubated with C11-BODIPY (581/591) (1 µM) for 30 min at 37°C in an incubator before trypsinization. Subsequently, cells were resuspended in 500 µL of fresh PBS (Gibco), strained through a 35-µM cell strainer (Falcon tube with cell strainer CAP) and analyzed by flow cytometer.
Instrument	Flow cytometer (FACS Canto II, BD Biosciences)
Software	Data analysis was conducted using the BD FACSVerseTM flow cytometer and FlowJo Software.
Cell population abundance	Cells strained through a 35-µM cell strainer (Falcon tube with cell strainer CAP). At least 30,000 cells in one tube and 10,000 cells were analyzed per sample.
Gating strategy	Gating was performed based on identifying a distinct population in FSC vs SSC plots.

- [x] Tick this box to confirm that a figure exemplifying the gating strategy is provided in the Supplementary Information.
